# The *Chlamydia* M278 Major Outer Membrane Peptide Encapsulated in the Poly(lactic acid)-Poly(ethylene glycol) Nanoparticulate Self-Adjuvanting Delivery System Protects Mice Against a *Chlamydia muridarum* Genital Tract Challenge by Stimulating Robust Systemic and Local Mucosal Immune Responses

**DOI:** 10.3389/fimmu.2018.02369

**Published:** 2018-10-15

**Authors:** Richa Verma, Rajnish Sahu, Saurabh Dixit, Skyla A. Duncan, Guillermo H. Giambartolomei, Shree R. Singh, Vida A. Dennis

**Affiliations:** ^1^Center for NanoBiotechnology Research, Alabama State University, Montgomery, AL, United States; ^2^Instituto de Inmunología, Genética y Metabolismo (INIGEM), CONICET, Universidad de Buenos Aires, Buenos Aires, Argentina

**Keywords:** *Chlamydia*, nanovaccine, PLA-PEG nanoparticles, IFN-γ, neutralizing antibodies, mucosal IgA

## Abstract

Recently, we reported that our PPM chlamydial nanovaccine [a biodegradable co-polymeric PLA-PEG (poly(lactic acid)-poly(ethylene glycol))-encapsulated M278 peptide (derived from the major outer membrane protein (MOMP) of *Chlamydia*)] exploits the caveolin-mediated endocytosis pathway for endosomal processing and MHC class II presentation to immune-potentiate *Chlamydia*-specific CD4^+^ T-cell immune effector responses. In the present study, we employed the *Chlamydia muridarum* mouse infection model to evaluate the protective efficacy of PPM against a genital tract challenge. Our results show that mice immunized with PPM were significantly protected against a homologous genital tract challenge evidently by reduced vaginal bacterial loads. Protection of mice correlated with enhanced *Chlamydia*-specific adaptive immune responses predominated by IFN-γ along with CD4^+^ T-cells proliferation and their differentiation to CD4^+^ memory (CD44^high^ CD62L^high^) and effector (CD44^high^ CD62L^low^) T-cell phenotypes. We observed the elevation of M278- and MOMP-specific serum antibodies with high avidity in the ascending order IgG1 > IgG2b > IgG2a. A key finding was the elevated mucosal IgG1 and IgA antibody titers followed by an increase in MOMP-specific IgA after the challenge. The Th1/Th2 antibody titer ratios (IgG2a/IgG1 and IgG2b/IgG1) revealed that PPM evoked a Th2-directed response, which skewed to a Th1-dominated antibody response after the bacterial challenge of mice. In addition, PPM immune sera neutralized the infectivity of *C. muridarum* in McCoy cells, suggesting the triggering of functional neutralizing antibodies. Herein, we reveal for the first time that subcutaneous immunization with the self-adjuvanting biodegradable co-polymeric PPM nanovaccine immune-potentiated robust CD4^+^ T cell-mediated immune effector responses; a mixed Th1 and Th2 antibody response and local mucosal IgA to protect mice against a chlamydial genital tract challenge.

## Introduction

*Chlamydia trachomatis* is an obligate, Gram-negative bacterium residing in the human genital tract and the leading bacterial sexually-transmitted infections worldwide (1). The pathogen initially targets the endo-cervical or urethral epithelium (2), but the infection is extremely concerning in females as the genital tract ascending infections may lead to pelvic inflammatory disease (PID), tubal factor infertility, ectopic pregnancy, and chronic pelvic pain (1, 3). In 2016, about 1.5 million cases of chlamydial infections were reported in the United States, while more than 100 million new cases are reported globally per year (4). Antibiotics are only effective during the early, but not established stages of infection, which strongly predicates the need for an efficacious vaccine to control the myriad of infections and complications associated with the disease (5–7).

Despite decades of efforts and trials to develop a vaccine against *Chlamydia*, there is yet no FDA approved vaccine for humans (8). Unsuccessful strategies of live, inactivated, or attenuated bacteria (9–11) as vaccine targets have prompted a switch toward subunit-based vaccine candidates (4, 6, 7, 12–20). Prominent amongst these is the major outer membrane protein (MOMP) of *Chlamydia* that is highly enriched with multiple T- and B-cell epitopes, expressed throughout the bacterium developmental cycle (6, 21). Reports have shown that MOMP triggers enhanced immune responses and neutralizing antibodies (22), which makes it a promising vaccine candidate against *Chlamydia* (23).

Administering native MOMP with adjuvants elicited strong immune responses that provided protection against a chlamydial challenge in mice (19, 24, 25) and non-human primates (26, 27). However, issues with the stability and scaling of native MOMP for vaccine development (4) shifted the focus toward recombinant MOMP (6, 28–31) and its T- and B-cell epitopes-based peptides (32–40). Studies of recombinant MOMP with the adjuvant, DDA/MPL (dimethyldioctadecylammonium bromide/monophosphoryl lipid A) in combination with chlamydial Pmps (7), CAF01 and CAF09 (19), TLR agonists (30, 41) or cholera toxin subunits (16, 28) showed significant protection against *Chlamydia* infection in mice. However, subunit vaccines against *Chlamydia* lacked complete or long-lasting protection (7) probably due to lack of suitable adjuvant (42) to bolster mucosal immune responses (28, 43) and/or efficient delivery systems (40).

We have generated a recombinant peptide of *C. trachomatis* MOMP consisting of small gene fragments containing T-cell epitopes (278–370 aa) and designated it as M278 (34). Immunogenicity studies with M278 in the absence of an adjuvant showed that a mixed Th1/Th2 response was evoked in mice, indicating the immunogenic nature of M278 and its potential to be used as a vaccine candidate against *Chlamydia* (34). We further reported that M278 encapsulated within the biodegradable co-polymeric PLA-PEG [poly (lactic acid)-poly (ethylene glycol)] nanoparticles potentiated robust *Chlamydia*-specific antibody and cellular adaptive immune responses in immunized mice (36). These encouraging results presumably were attributed to the self-adjuvanting and slow-releasing properties (40, 44) of the PLA-PEG nanoparticles vaccine-delivery system. Recently, we showed that PLA-PEG-encapsulated M278 immunopotentiation of immune effector responses is mediated by endosomal processing and MHC class II-dependent presentation to trigger robust *Chlamydia*-specific immune effectors mediated by CD4^+^ T cells (40).

Based on the above findings, we hypothesized that the T-cell epitopes-enriched M278 peptide formulated with PLA-PEG would be efficacious in providing protection against a chlamydial genital challenge in immunized mice (36). In the present study, we evaluated the protective efficacy of PLA-PEG-encapsulated M278 in immunized female mice against a chlamydial genital challenge. We report that immunization of mice with PLA-PEG-encapsulated M278 triggered significant cellular as well as systemic and mucosal antibody responses to protect mice against a *C. muridarum* vaginal challenge. Moreover, enhanced secretion of mucosal IgA implied that, it might play a role in protecting mice against vaginal challenge by boosting mucosal immunity. Our results are presented and discussed regarding the effectiveness of PLA-PEG as a delivery system, and PLA-PEG-encapsulated M278 as an attractive nanovaccine candidate against a chlamydial genital tract challenge.

## Materials and methods

### Bacteria and reagents

*Chlamydia muridarum* [strain Nigg II; previously called *C. trachomatis* mouse pneumonitis (MoPn) biovar] expressed as inclusion forming units (IFU/mL) was purchased from Virusys Corporation (Taneytown, MD). The mouse-derived McCoy fibroblasts cell line and DMEM with high glucose and L-Glutamine were purchased from American Type Culture Collection (ATCC; Manassas, VA). PEG-b-PLA diblock polymer (polyethylene glycol; MW 10,000 and polylactic acid, MW 5,000) was purchased from Polysciences Incorporation (Warrington, PA). Polyvinyl alcohol (PVA), ethyl acetate, and mitomycin-C were purchased from Sigma-Aldrich (St Louis, MO). ELISA MAX™ Deluxe kit for IFN-γ, IL-6, and IL-2 were purchased from BioLegend (San Diego, CA). RPMI 1640 with GlutaMax™ and HEPES, heat-inactivated fetal bovine serum (FBS), Goat serum, ACK lysing reagent and antibiotic-antimycotic were all purchased from Life Technologies (Grand Island, NY). Anti-CD 90.2 magnetic beads and MACS columns were purchased from Miltenyi Biotech (Auburn, CA). CellTrace™ CFSE (carboxyfluorescein succinimidyl ester) cell proliferation assay kit (C34554) and PathoDx™ Chlamydia culture confirmation kit were purchased from ThermoFisher Scientific (Rockford, IL). The fluorochrome-conjugated antibodies: CD3-APC-Cy7 (BD:560590), CD4-PerCP-Cy5.5 (BD:550954), CD62L-APC (BD:553152), CD44-PE (BD:553134), and Opti-EIA sets for IL-10 and TNF-α were obtained from BD-Biosciences (San Jose, CA). Depo-Provera was purchased from Pfizer (New York, NY). Cycloheximide was purchased from EMD Biosciences (La Jolla, CA).

### Formulation of the M278 nanovaccine

A recombinant peptide (M278) derived from the major outer membrane protein (MOMP) of *C. trachomatis* was cloned into pET-32 vector, expressed in *E. coli* BL21 (DE3) cells and purified using His-Bind Columns as previously described by us (34). The M278 peptide was encapsulated in PLA-PEG [poly(lactic acid)-poly (ethylene glycol)] biodegradable nanoparticles using a modified water/oil/water double emulsion evaporation technique to obtain PLA-PEG-M278 (PPM) as reported (36). An equivalent volume of sterile PBS as used for M278 was similarly encapsulated in PLA-PEG to obtain PLA-PEG-PBS (PPP) to serve as a negative control.

### Mice

Female 4–6 weeks-old BALB/c mice were purchased from Charles River Laboratory (Raleigh, NC) and acclimatized for 2 weeks prior to all experimental procedures. The animal studies were performed following a protocol approved by the Alabama State University's Institutional Animal Care and Use Committee (IACUC). Mice were housed under standard pathogen-free and controlled environmental conditions and provided with food and water *ad libitum*.

### Mice immunization and challenge

Mice were divided into three experimental groups (11 mice/group) for immunization (36) and challenge with *C. muridarum* (Cm). Groups of mice received three subcutaneous immunizations at 2 week intervals with M278 and nanoparticles (PPP or PPM). The PPM mice each received 50 μg/100 μL of encapsulated-M278 in sterile PBS (8 mg total weight); those in the PPP group each received an equivalent weight of PBS encapsulated in PLA-PEG. Mice in the M278 group received 50 μg/100 μL of purified M278 in sterile PBS. Two-weeks following the last immunization (day 42), mice (6 mice/group) were sacrificed to collect spleen, serum and cervico-vaginal wash samples for cellular and antibody analyses, respectively. For the challenge study, immunized mice (5 mice/group) were injected subcutaneously with 2.5 mg of medroxyprogesterone acetate (Depo-Provera) 2 weeks after the last immunization, follow 1 week later by an intravaginal challenge with 1 × 10^5^ IFU of Cm in sucrose-phosphate-glutamic acid (SPG) buffer (25). All groups of challenged mice were sacrificed 3 weeks post-challenge to collect serum and vaginal wash samples for antibody analyses.

### Quantification of *Chlamydia* vaginal load

Cervico-vaginal swabs were collected in SPG buffer at 3 days intervals up to 3 weeks after the challenge infection and stored at −80°C for quantification of the chlamydial vaginal load according to published methods (25). Briefly, McCoy cells were propagated in DMEM and seeded at (5 × 10^4^/well) in 96-well cell culture plates. Confluent cell monolayers were inoculated with cervico-vaginal swab suspensions containing 0.5 μg/mL cycloheximide, centrifuged at 750 g for 1 h at room temperature (RT) and then incubated for 2 h at 37°C in a 5% CO_2_ humidified atmosphere. Thereafter, the medium was replaced with fresh medium containing 0.5 μg/mL cycloheximide and incubated for 30 h at 37°C in a 5% CO_2_ humidified atmosphere. The cells were washed, fixed in 95% ethanol and stained with PathoDx™ *Chlamydia* Culture confirmation kit, and inclusions were visualized and counted using a Nikon confocal microscope.

### T-cells stimulation

T-cells stimulation was performed as described (36). In brief, spleens were collected from groups of immunized and immunized-challenged mice and pooled per group in RPMI 1640 supplemented with 10% FBS and antibiotic-antimycotic. Single spleen cell suspensions were obtained, filtered through 40-μm nylon mesh strainer, and washed after red blood cells lyses using ACK lysing solution. The cells were incubated with anti-CD 90.2-conjugated magnetic beads and total purified T-cells were isolated by positive selection over MACS columns. Naïve single spleen cell suspensions were treated with mitomycin-C (25 μg/mL) for 30 min at 37°C in a 5% CO_2_ humidified atmosphere followed by washing four times in RPMI 1640 (300 g for 10 min) and used as antigen presenting cells (APCs) for T-cell stimulation. Purified T-cells (1 × 10^6^) and APCs (1 × 10^6^) co-cultures were stimulated with M278 (2.5 μg/mL) in round bottom-polypropylene tissue culture tubes and incubated for 120 h at 37°C in a 5% CO_2_ humidified atmosphere. For some experiments, co-cultures were also stimulated with 1 × 10^5^ IFU of UV-inactivated Cm (UV-Cm) or with Concanavalin A (5 μg/mL). Cell-free culture supernatants were collected by centrifugation and stored at −80°C until used. The selected 2.5 μg/mL concentration of M278 was based on previous titration studies (40).

### Assessments of T-cells proliferation and memory and effector phenotypes

The CFSE proliferation assay was performed as recently reported (40). Briefly, purified T-cells isolated from splenocytes of immunized mice were labeled with 5 μM CFSE by incubating cells for 20 min at 37°C in a 5% CO_2_ humidified atmosphere. Labeled T-cells (0.5 × 10^6^ cells) were co-cultured with APCs (0.5 × 10^6^ cells) and stimulated with UV-Cm (1 × 10^5^) in tissue culture tubes and incubated for 120 h at 37°C in a 5% CO_2_ humidified atmosphere. After incubation, the cells were harvested and stained using CD3-APC-Cy7, CD4-PerCP-Cy 5.5, CD62L-APC, and CD44-PE to quantify T-cells proliferation, memory and effector phenotypes. Stained cells were washed, fixed and data were acquired on a BD LSR II flow cytometer and analyzed using FCS Express 6 software (De Novo Software, Glendale, CA). Gating on CFSE-labeled total T-cells was used for the selection of CD3^+^CD4^+^ T-cell populations. Then, histogram fluorescence intensities were used to quantify the proliferating and resting cells amongst the total CFSE^+^CD3^+^CD4^+^ T-cells.

### Quantification of cytokines

Cytokines (IL-6, TNF- α, IFN-γ, IL-2, IL-10) were quantified in cell free-culture supernatants using specific ELISA kits as described (40). The experiments were repeated at least two times and each sample was run in triplicates.

### Serum and mucosal antibody responses

Serum and vaginal washed samples were collected from each group of mice and pooled per group for the detection of specific antibody isotypes (IgG, IgG1, IgG2a IgG2b, and IgA) as previously described (6, 28, 36). To determine the antigen-specific antibodies, the ELISA plates were coated with 100 μL (1 μg/mL) of purified proteins (M278 or MOMP) overnight at 4°C followed by washing with PBST (PBS-Tween 20) and blocking in 3% non-fat dry milk. The recombinant MOMP used in this study was cloned, expressed, and purified as previously described by us (6). The ELISA plates were washed again with PBST and serum or cervico-vaginal wash samples were serially diluted two-fold starting at 1:4,000 (serum IgG1), 1:500 (serum IgG2a, IgG2b), 1:25 (vaginal wash IgG1, IgG2a, IgG2b) and 1:5 (wash IgA) and added to the plates. After washing, antigen-specific IgG1, IgG2a, IgG2b, and IgA antibodies were detected using isotype-specific HRP-conjugated goat anti-mouse antibodies and TMB substrate. The endpoint titer was considered to be the last sample dilution with readings higher than the mean + 5 standard deviations of the negative control samples (serum IgG isotypes and vaginal wash IgG1) or the mean + 3 standard deviations of the negative control vaginal wash samples (vaginal wash IgA). All samples were run in duplicates and experiments were repeated at least two times.

### Serum-immunoglobulin avidity

To determine the avidity index, the ELISA plates were coated with 100 μL (1 μg/mL) purified proteins (M278 or MOMP) overnight at 4°C followed by washing with PBST (PBS-Tween 20) and blocking in 3% non-fat dry milk. Optimally diluted sera samples (1:50, 1:100, 1:200 and 1:400) were added to wells of the M278- or MOMP-coated plates in parallel (2 sets per plate) and incubated for 2 h at RT. After washing with PBST, one set for each serum sample was treated with urea (8M in PBST) and the other set was treated with PBST for 5 min at RT. After washing, antigen-specific IgG1 (Th2) and IgG2b (Th1) were detected using isotype-specific HRP-conjugated goat anti-mouse antibodies and TMB substrate. IgG2b was used since mice produced higher IgG2b than IgG2a antibodies. The avidity index (AI) was calculated using the formula below (45).

(1) $$\begin{array}{r} \text{Avidity Index}\left(\text{\%}\right)=\left[\left(\text{OD with urea}\right)/\left(\text{OD without urea}\right)\right]\times 100 \end{array}$$

### *In vitro* serum neutralization assay

The assay was performed using mouse McCoy fibroblasts for infection with Cm. Briefly, Cm elementary bodies (EBs) at 500 IFU per well in a 96-well plate (46) were treated with optimally diluted serum (1:100) from groups of immunized mice and incubated for 30 min at 37°C on a slowly rocking platform. Treated-EBs were then added in triplicates to confluent monolayers and centrifuged at 750g for 1 h, followed by incubation for 2 h at 37°C in a 5% CO_2_ humidified atmosphere. The media were replaced with fresh DMEM containing 10% FBS and 0.5 μg/mL cycloheximide and then incubated for 30 h (47). EBs without serum treatment served as positive control and naïve serum was used as negative control. After incubation, the plates were fixed with 95% ethanol and stained using PathoDx™ Chlamydia culture confirmation kit. Inclusions were imaged and counted using a Nikon confocal microscope. Imaging was done for 10 different fields per well using a 10x objective lens.

### Statistical analysis

Data were analyzed by the two-tailed unpaired Student's *t*-test followed by Welch's correction, or one- or two-way analysis of variance (ANOVA) followed by Tukey's Post-test using GraphPad Prism 6 Software (GraphPad Software, Inc., CA, USA). *P*-values ≤ 0.05 were considered statistically significant.

## Results

### Protection against a live *C. muridarum* genial tract challenge

**Figure 1:**
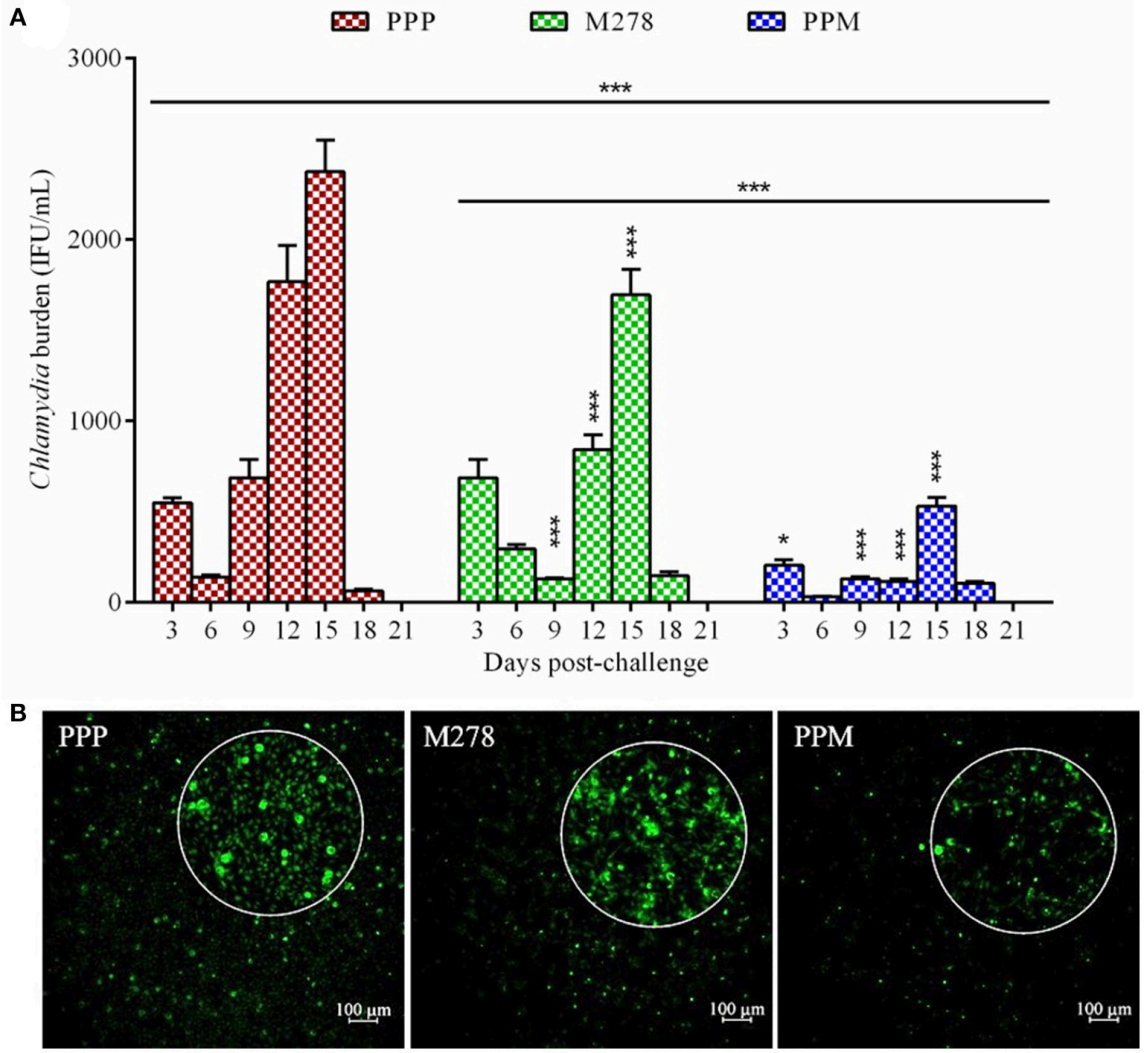
Quantification of *C. muridarum* burden from vaginal cultures of mice following a genital tract challenge infection. Groups of mice received three subcutaneous immunizations at 2 weeks intervals with either bare M278, PPM, or the PPP control. Each mouse per group was challenged intravaginally with 1 × 10^5^ IFU (inclusion forming units) of *C. muridarum* 3 weeks following the last immunization. Cervico-vaginal swabs were collected for a period of 3 weeks post-challenge for quantification of IFUs after propagation in McCoy mouse fibroblasts. Each bar represents the mean ± standard deviation of IFU counts (IFU/mL) for each group of mice at the indicated time-points **(A)**. Immunofluorescence microscopy visualization of *C. muridarum* IFU (green) in fibroblasts (with magnified view) exposed to cervico-vaginal swabs from immunized mice at the 12 day post-challenge time-point **(B)**. Statistical analysis in **(A)** was performed using ANOVA followed by Tukey's Post-test. Significant differences in IFU counts between the M278 and PPM immunized groups and the PPP immunized control group were considered at ^***^P < 0.001 and ^*^P < 0.05. PPP (PLA-PEG-PBS immunized group); M278 (bare M278 immunized group); PPM (PLA-PEG-M278 immunized group).

We recently reported that the PPM nanovaccine immune-potentiates innate and adaptive immune responses in immunized mice (36, 40). Given that PPM triggered adaptive cellular and humoral immune response correlates of *Chlamydia* protective immunity in mice, we hypothesized that PPM would be efficacious in providing protection against a chlamydial genital challenge infection. To investigate this, groups of mice were prophylactically immunized via the subcutaneous route three times at 2 weeks intervals with bare M278, PPM or the PPP control. Three-weeks following the last immunization, each mouse was challenged intravaginally with Cm (1 × 10^5^ IFU) and cervico-vaginal swabs were collected at different post-infection time-points to quantify the recovered IFU. Our results as shown in Figure 1A revealed that subcutaneous immunization of mice with PPM provided significant (*P* < 0.001) protection against the challenge by reduced bacterial loads, respectively of ~ 63, 76, 81, 93, and 77% on days 3, 6, 9, 12, and 15 post-challenge over those of the PPP control group. The bare M278 immunized mice also were significantly (*P* < 0.001) protected against the challenge as compared to the PPP control group but overall with lesser reduction in their bacterial loads of ~ 81, 52, and 29%, respectively on days 9, 12, and 15 post-challenge.

Of interest, was the significant (*P* < 0.001) differences in overall bacterial load and at individual time-points on days 3, 12, and 15 post-challenge observed between bare M278- and PPM-immunized mice, suggesting that PPM immunization confers an enhanced protection of mice (Figure 1A). Additionally, immunofluorescence microscopy images provide direct confirmation of the lesser number of inclusions in cells exposed to swabs collected from PPM as compared to those from PPP and M278 immunized mice (Figure 1B). By day 21 post-challenge the infection was cleared spontaneously in all mice; a finding that has been reported by other investigators (21, 48, 49). These encouraging results provide the first evidence that the PPM nanovaccine is efficacious in providing protection against a homologous chlamydial genital challenge in mice.

### Cytokines produced by T-cells from immunized and immunized-challenged mice

**Figure 2:**
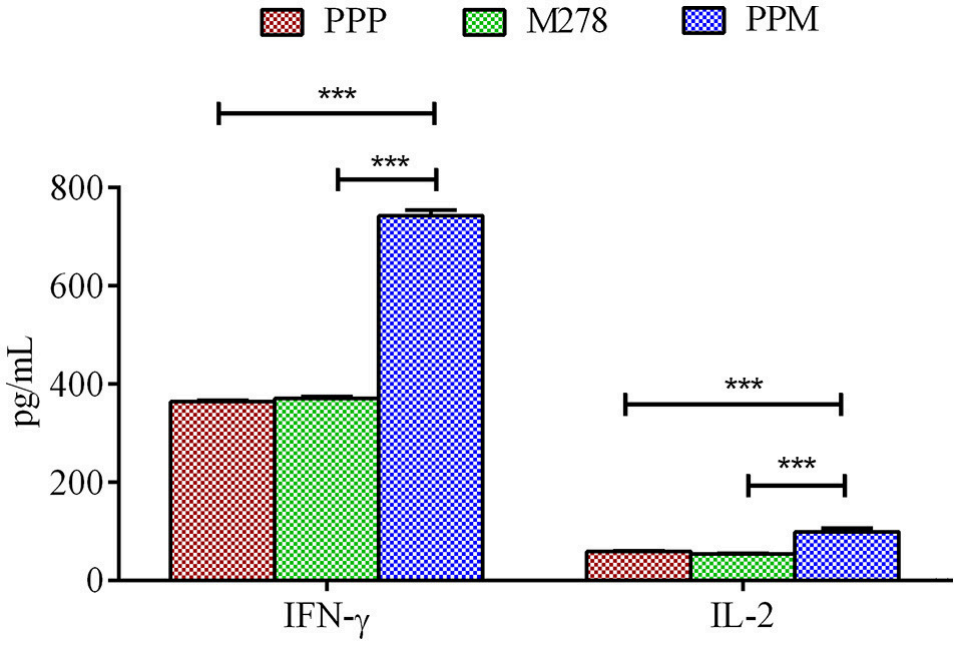
Cytokines production by T-cells from immunized mice. Purified T-cells (1 × 10^6^) and APCs (1 × 10^6^) were stimulated with purified M278 (2.5 μg/mL) and incubated for 120 h at 37°C in a 5% CO_2_ humidified atmosphere. Cell-free supernatants were collected by centrifugation and used to quantify cytokines (IFN-γ and IL-2) using specific ELISA. Each bar represents the mean ± standard deviation of triplicate samples. Significance was considered at ***P < 0.001. PPP (PLA-PEG-PBS immunized mice); M278 (bare M278-immunized mice); PPM (PLA-PEG-M278)-immunized mice.

Acquired immunity to *Chlamydia* is mediated by CD4^+^ T-cells producing Th1 type cytokines such as IFN-γ, which are considered correlates of protective immunity in human *Chlamydia* infections (50). Consequently, we next assessed the production levels of Th1 and Th2 cytokines by immune T-cells from PPP, PPM-, and M278-immunized mice. Purified T-cells and APCs co-cultures were stimulated with M278, and Th1 (IFN-γ, IL-2) and Th2 (IL-10) cytokines were quantified in cell-free supernatants using specific ELISA. As illustrated in Figure 2, T-cells from PPM-immunized mice produced significantly more IFN-γ (*P* < 0.001) and IL-2 (*P* < 0.001) than those from the bare M278 and PPP-immunized mice. No differences were seen in the IFN-γ and IL-2 responses between the bare M278 and PPP mice. Moreover, we did not observe any difference in the secretion levels of the Th2 cytokine, IL-10 (~200 pg/mL) between groups of mice, thereby confirming that the Th1 cytokine, IFN-γ was predominantly induced by the PPM nanovaccine in immunized mice. The IFN-γ responses to Concanavalin A (a T-cell mitogen) stimulation of T-cells from immunized mice, show that the secretion of IFN-γ is specifically from T-cells and not the mitomycin-C treated APCs. (Figure S2).

*Chlamydia* vaccine trials are often associated with enhanced inflammatory responses post-vaccination or re-infection after vaccination due to incomplete immunity engendered by certain vaccines candidates as reviewed recently (4). Therefore, we evaluated the secretion levels of the M278-specific inflammatory cytokines, IL-6 and TNF-α, and the immuno-protective cytokine, IFN-γ by T-cells from immunized mice 3 weeks following a chlamydial challenge. The results showed that low IL-6 and negligible TNF-α levels were produced by T-cells from the PPM mice in comparison to an enhanced IFN-γ response. Also, these cytokines were not robustly produced by T-cells from the M278 and PPP mice, except a higher IFN-γ response in the PPP mice after challenge, suggesting the potentiating property of the PLA-PEG nanoparticles (Figure S1).

### *Chlamydia*-specific T-cell proliferation, memory and effector phenotypes

**Figure 3:**
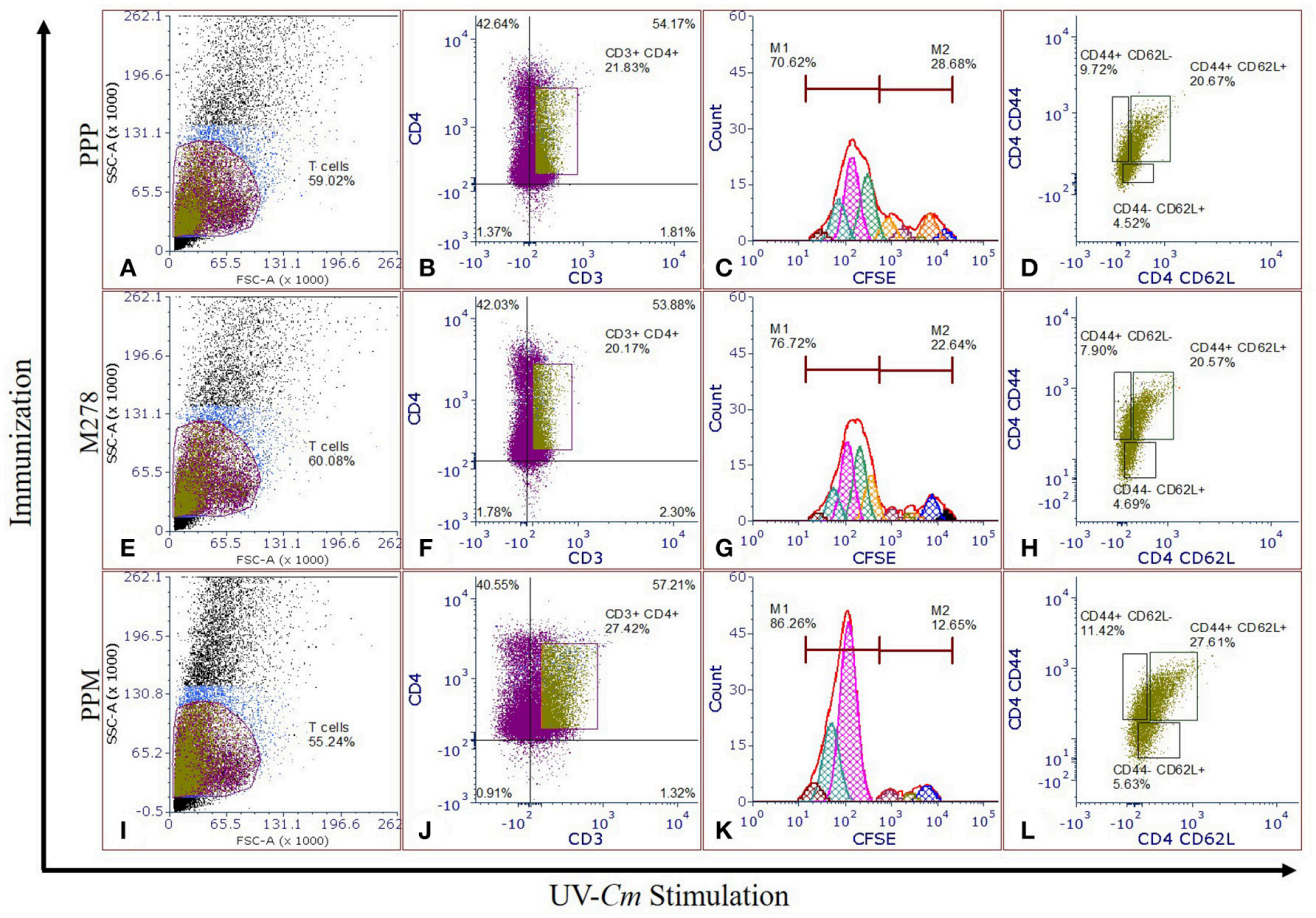
*Chlamydia*-specific T-cells proliferation, and memory and effector T-cells phenotypes. CFSE labeled T-cells from PPP **(A–D)**, M278 **(E–H)**, and PPM **(I–L)** immunized mice were co-cultured with naïve APCs, stimulated with 1 × 10^5^ IFU of UV-Cm followed by incubation for 120 h. Proliferating T-cells (CFSE^+^CD3^+^), and memory and effector phenotypes in proliferating T-cells populations were quantified by staining for CD3, CD4, CD44, and CD62L. Histograms **(C,G,K)** of CFSE fluorescence intensity to quantify CFSE^+^CD3^+^CD4^+^ proliferating (M1) and resting (M2) T-cells, and dot plots showing the expression of CD44 and CD62L **(D,H,L)** were derived by gating on CFSE^+^CD3^+^CD4^+^ stimulated T-cells **(B,F,J)** from PPP **(A–D)**, M278 **(E–H)**, and PPM **(I–L)** immunized mice. PPP (PLA-PEG-PBS immunized group); M278 (bare M278 immunized group); PPM (PLA-PEG-M278 immunized group); UV-Cm (UV-inactivated Cm).

Proliferating T-cells are indicative of a mounting cellular adaptive immune response and induction of antigen-specific memory and effector T-cell responses are the prerequisites of an efficacious vaccine; which provide immune surveillance in tissues. CD4^+^ memory and effector T-cells play crucial roles in vaccine-induced immunity against *Chlamydia*. As such, we assessed whether PPM triggered enhanced T-cells activation and immune effectors to protect mice against a chlamydial genital challenge. CFSE-labeled T-cells were co-cultured with naïve APCs and stimulated with UV-Cm for 120 h to evaluate T-cell proliferation as well as memory and effector phenotypes from the PPP (Figures 3A–D), M278 (Figures 3E–H) and PPM (Figures 3I–L) immunized mice. We specifically selected UV-Cm as the stimulant for this study to assess the impact of the whole bacteria in recalling memory and effector immune responses. Our results demonstrate that stimulated CD4^+^ T-cells from all groups of mice proliferated over several generations; but their percentages of resting CD4^+^ T-cells (M2 population) were low, respectively 12.49, 22.64, and 28.66% for the PPM, M278 and PPP immunized groups of mice. Contrastingly, proliferating CD4^+^ T-cells (M1 population) from PPM-immunized mice were higher in magnitude (86.26%) as compared to the M278 (76.72%) and PPP (70.62%) mice (Figures 3C,G,K), suggesting enhanced activation of CD4^+^ T-cells by the PPM nanovaccine.

We further quantified both memory (CD44^high^ and CD62L^high^) and effector (CD44^high^ and CD62L^low^) phenotypes by gating on the CFSE^+^CD3^+^CD4^+^ T-cell populations. Our results revealed that 27.61% of the proliferating CD4^+^ T-cells from PPM-immunized mice differentiated into the memory phenotype (CD44^high^ and CD62L^high^) as compared with 20.57 and 20.67%, respectively for M278 and PPP-immunized groups of mice. Moreover, an enhancement in CD4^+^ effector T-cell phenotypes (CD44^high^ and CD62L^low^) was observed in the PPM group (11.42%) in comparison to the M278 (7.90%) and PPP (9.72%) groups (Figures 3L,H,D). The increased percentages of effector T-cell phenotypes in the PPP group as compared to the M278 group could likely result from fewer memory cells, while the enhanced naïve (CD44^low^ and CD62L^high^) phenotype in the PPM-immunized mice may be due to the increased percentage of CD3^+^CD4^+^ proliferating T-cells. Taken together, our results demonstrate that T-cells from PPM-immunized mice exhibited enhanced CD3^+^CD4^+^ chlamydial-specific proliferation and memory and effector T-cells, which are indicative of PPM stimulating a more effective response to a subsequent bacterial encounter.

### Production of antigen-specific serum antibodies in immunized mice

**Figure 4:**
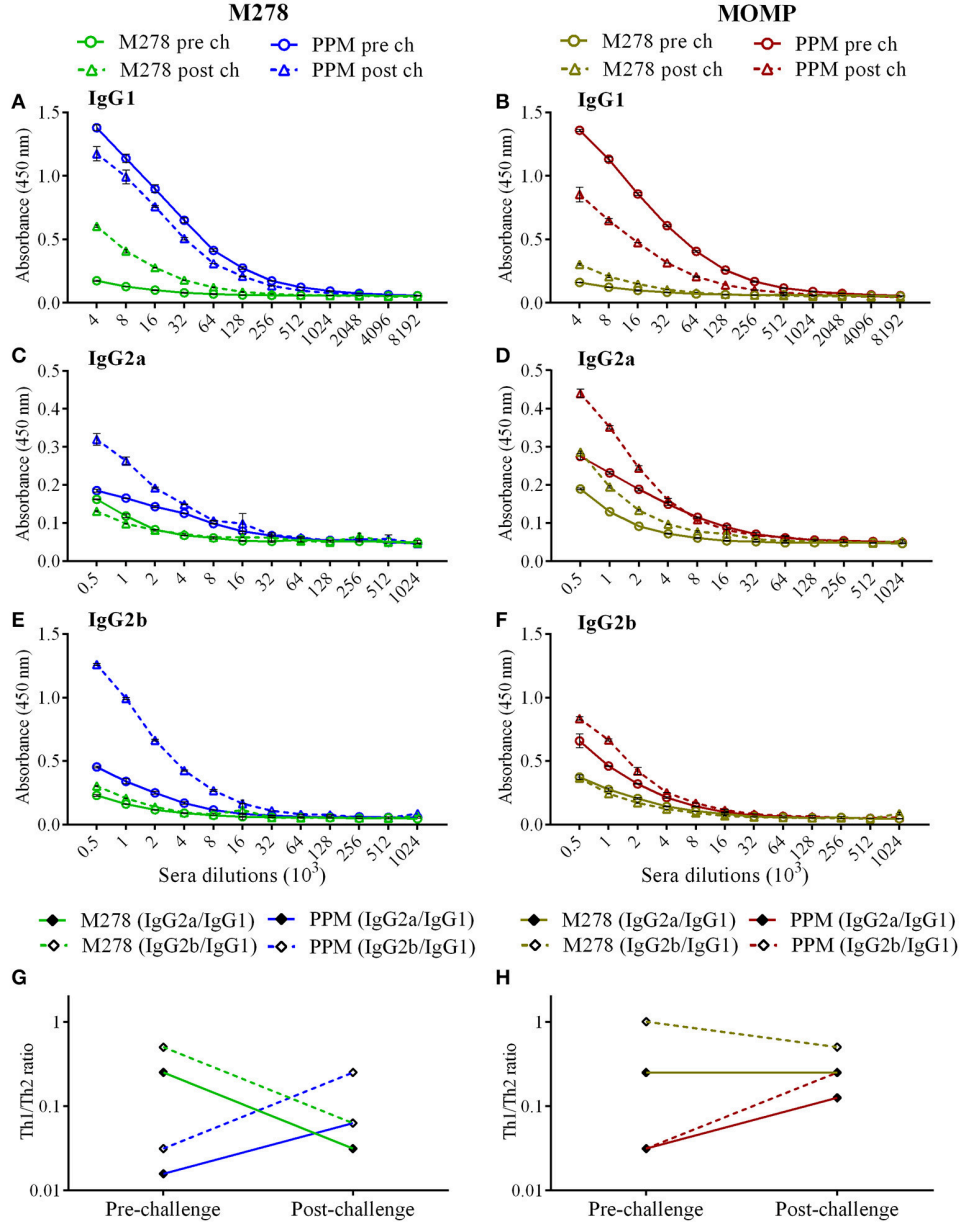
Antigen-specific serum antibodies production in immunized mice. Groups of mice received three subcutaneous immunizations at 2 weeks intervals with either bare M278 or PPM. Three-weeks following the last immunization, each mouse was challenged intravaginally with 1 × 10^5^ IFU of *C. muridarum*. M278-specific IgG1 **(A)**, IgG2a **(C)**, IgG2b **(E)**, and MOMP-specific IgG1 **(B)**, IgG2a **(D)**, and IgG2b **(F)** antibodies were measured by ELISA in pooled sera 2 weeks after the third immunization (pre-challenge) and 3 weeks after the challenge infection (post-challenge). Sera were diluted at a two-fold serial dilution to determine the end-point antibody isotype titers. M278-specific **(G)** and MOMP-specific **(H)** Th1/Th2 (IgG2a/IgG1 and IgG2b/IgG1) antibody ratios were calculated using the endpoint titers. Each data point represents the mean ± standard deviation of duplicate samples and each experiment was repeated at least two times. M278 (bare M278 immunized group); PPM (PLA-PEG-M278 immunized group); pre ch (pre-challenge); post ch (post-challenge).

It is well-documented that the humoral and cellular immunity arms of the immune system play significant roles in bacterial clearance and protection from re-infection (4). The noteworthy efficacious protection provided by the PPM nanovaccine (Figure 1) prompted further investigations into the correlation of the observed protective immunity in immunized mice with that of specific-antibody immune responses. In addition, we assessed how a *C. muridarum* challenge might impact pre-existing antibody responses in immunized mice. Thus, antibody ELISAs were conducted using pooled sera from immunized (pre-challenge) and immunized-challenge (post-challenge) mice against the specific M278 peptide and the parent protein (MOMP). We observed that mice immunized with PPM produced higher pre-and post-challenge antigen-specific Th1 (IgG2a and IgG2b) and Th2 (IgG1) antibodies as compared to bare M278 (Figure 4, Table 1). PPM-immunized mice produced a 64-fold higher antigen-specific IgG1 titer in comparison to that of the M278-immunized mice. However, after challenge, the bare M278 immunized mice increased their antigen-specific IgG1 antibody titer, while a reduced IgG1 titer was observed for the PPM-immunized mice as evidenced by only 4- and 8-fold increases for M278- and MOMP-specific IgG1 titers over those of bare M278 (Figures 4A,B, Table 1). The titers of IgG1, IgG2a, and IgG2b antibodies in the PPP-immunized mice remained below the cut-off level, however low antibody titers were detected following the bacterial challenge (Table 1).

**Table 1:** Antigen-specific serum-antibody endpoint titers of immunized mice.

		**PPP**		**M278**		**PPM**	
**Antibody isotypes**	**Antigen**	**Pre-challenge**	**Post-challenge**	**Pre-challenge**	**Post-challenge**	**Pre-challenge**	**Post-challenge**
IgG1	M278	–	4,000	8,000	64,000	512,000	256,000
	MOMP	–	8,000	8,000	16,000	512,000	128,000
IgG2a	M278	–	2,000	2,000	2,000	8,000	16,000
	MOMP	–	4,000	2,000	4,000	16,000	16,000
IgG2b	M278	–	2,000	4,000	4,000	16,000	64,000
	MOMP	–	2,000	8,000	8,000	16,000	32,000

*PPP, PLA-PEG-PBS immunized group; M278, bare M278 immunized group; PPM, PLA-PEG-M278 immunized group. The endpoint titer was considered to be the last sample dilution with readings higher than the mean + 5 standard deviations of the negative control sera*.

Overall, IgG2a and IgG2b antigen-specific antibodies were lower than the IgG1 in sera of PPM- and M278-immunized mice (Figures 4C–F). Nevertheless, mice immunized with PPM still produced higher levels (2 to 8-fold) of IgG2a and IgG2b antigen-specific antibodies as compared to the M278 immunized mice (Figures 4C–F, Table 1.) Chlamydial challenge of PPM-immunized mice induced greater levels/titers of IgG2b antibodies specific to M278 (4-fold higher) and MOMP (2-fold higher). The pre- and post-challenge IgG2b antibody titers of PPM immunized mice were higher (4 to 16-fold) than the bare M278 mice (Figures 4E,F, Table 1).

Further analyses of the Th1/Th2 (IgG2a/IgG1 and IgG2b/IgG1) antibody ratios were conducted to unveil skewing patterns that may be predictive of protective immunity in immunized mice. As shown in Figures 4G,H, PPM-immunized mice had lower M278- and MOMP-specific IgG2a/IgG1 and IgG2b/IgG1 ratios, which are indicative of a predominant Th2 antibody response. But after challenge, an increase in the ratios toward a Th1-skewed pattern was observed in these mice. Conversely, the bare M278-immunized mice exhibited higher IgG2a/IgG1 and IgG2b/IgG1 ratios before the challenge, but these were reduced with a shift toward a Th2 pattern after a chlamydial challenge infection. Overall, these results reveal an insight into possible Th1and Th2 antibody-protective mechanisms afforded by PPM being predominated by a Th2 (IgG1) response pre-challenge, and a subsequent skewing toward Th1 (IgG2a and IgG2b) responses after a chlamydial challenge.

### Production of antigen-specific mucosal antibodies in immunized mice

**Figure 5:**
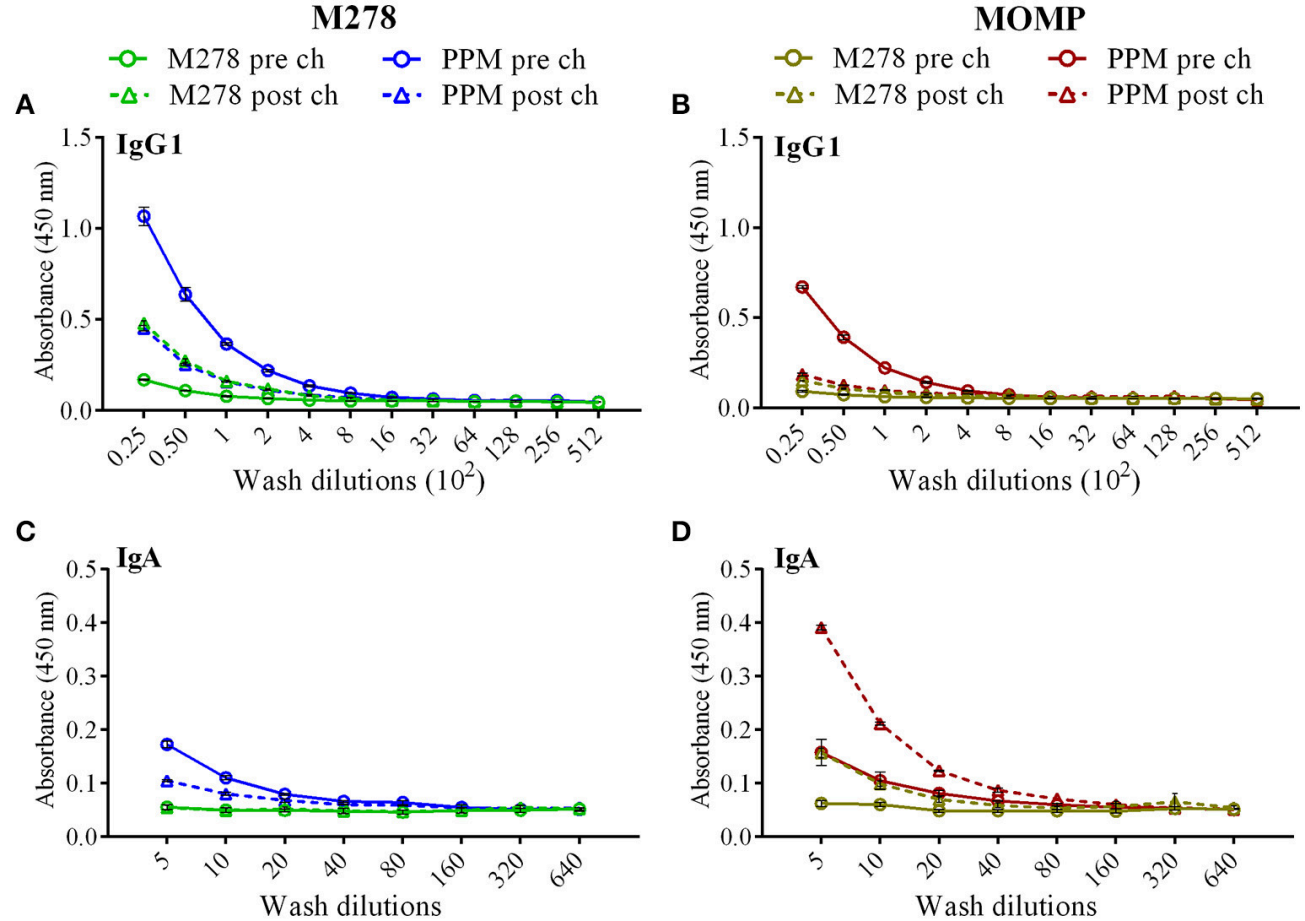
Antigen-specific mucosal antibodies in cervico-vaginal washes from immunized mice. Mice were immunized with bare M278 and encapsulated-M278 (PPM) and challenged intravaginally with *C. muridarum* as described above. M278-specific IgG1 **(A)**, MOMP-specific IgG1 **(B)**, M278-specific IgA **(C)** and MOMP-specific IgA **(D)** antibodies were measured by ELISA in the pooled vaginal wash samples of immunized mice before (pre-challenge) and 3 weeks following a *C. muridarum* challenge infection (post-challenge). Vaginal wash samples were diluted at a two-fold serial dilution to determine the end-point antibody isotype titers and each data point represents the mean ± standard deviation of duplicate samples. M278 (bare M278 immunized group); PPM (PLA-PEG-M278 immunized group); pre ch (pre-challenge); post ch (post-challenge).

As antibody isotypes in genital secretions play critical roles in preventing *Chlamydia* transmission, we continued to investigate whether the mucosal antibody responses were also elevated following immunization of mice with either bare M278, PPM, or PPP. ELISA was conducted using pooled vaginal wash samples from pre- and post-challenge immunized mice. Both M278- and MOMP-specific IgG1 responses were increased by 16-fold in animals immunized with PPM as compared to those immunized with bare M278 (Figures 5A,B, Table 2). Chlamydial challenge of the bare M278 immunized mice caused an increase of M278- and MOMP-specific IgG1 responses, while the bacterial challenge of the PPM mice down-regulated these antibody responses (Figures 5A,B, Table 2). Overall, PPP-immunized mice produced lower or equivalent specific IgG1 antibody titers than the bare M278 and PPM immunized mice pre- and post-challenge (Table 2). Mucosal antigen-specific IgG2a and IgG2b antibodies were low in both PPM- and M278-immunized mice (data not shown). Irrespective, calculations of the Th1/Th2 (IgG2b/IgG1 or IgG2a/IgG1) ratios for the vaginal wash samples depicted a similar skewed Th1/Th2 pattern (data not shown) as observed for the serum antibody ratios.

**Table 2:** Mucosal antibody endpoint titers of immunized mice.

		**PPP**		**M278**		**PPM**	
**Antibody isotypes**	**Antigen**	**Pre-challenge**	**Post-challenge**	**Pre-challenge**	**Post-challenge**	**Pre-challenge**	**Post-challenge**
IgG1	M278	25	100	200	800	3,200	1,600
	MOMP	100	200	100	400	1,600	800
IgA	M278	–	–	–	–	40	20
	MOMP	–	–	5	20	40	80

*PPP, PLA-PEG-PBS immunized group; M278, bare M278 immunized group; PPM, PLA-PEG-M278 immunized group. The endpoint titer was considered to be the last sample dilution with readings higher than the mean + 5 standard deviations for IgG1 and mean + 3 standard deviations for IgA of the negative control wash sample*.

Measurement of the mucosal IgA antibody in the cervico-vaginal wash samples revealed that both M278- and MOMP-specific IgA responses were higher in PPM-immunized mice as compared to the bare M278 mice (Figures 5C,D, Table 2). After receiving a bacterial challenge infection, M278-specific IgA decreased by 2-fold in the PPM mice (Figure 5C, Table 2). An interesting observation was the production of higher MOMP-specific IgA levels/titers in both PPM- and bare M278-immunized mice following the challenge infection (Figure 5D, Table 2). Immunization with PPP did not elicit an IgA response (Table 2. These findings provide a possible contributing role of mucosal antibody responses to the protection afforded in mice by the PPM nanovaccine against a Cm challenge.

### Antigen-specific serum IgG1 and IgG2b antibody avidity

**Figure 6:**
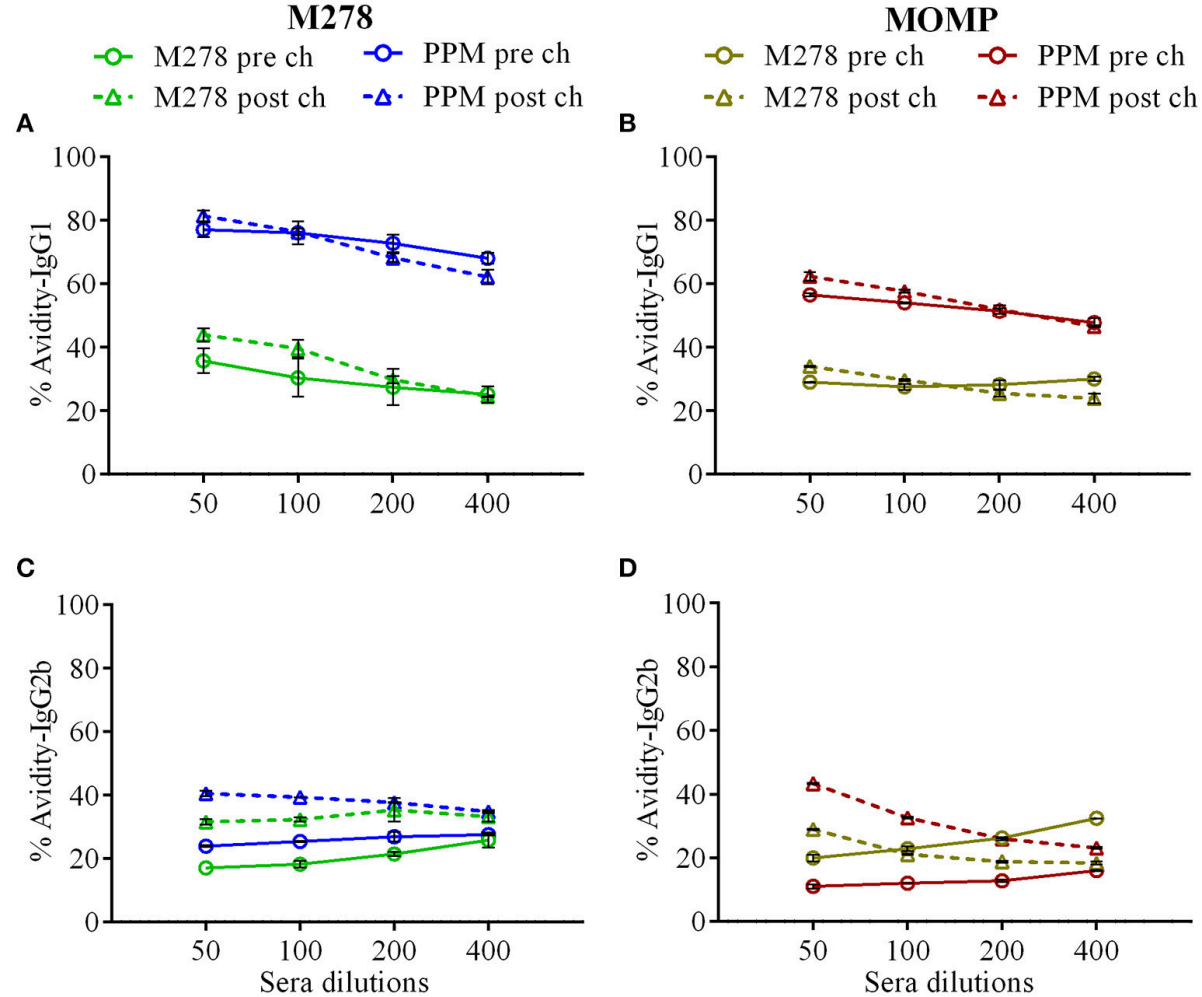
Avidity index of *Chlamydia*-specific serum IgG1 and IgG2b antibodies. Avidity-ELISA was conducted using pooled sera from immunized and *C. muridarum* challenged immunized mice to determine the avidity index (%) for M278-specific IgG1 **(A)**, MOMP-specific IgG1 **(B)**, M278-specific IgG2b **(C)** and MOMP-specific IgG2b **(D)** antibody isotypes. Each data point represents the mean ± standard deviation of duplicate samples. M278 (bare M278-immunized group); PPM (PLA-PEG-M278)-immunized group; pre ch (pre-challenge); post ch (post-challenge).

Given the enhanced antigen-specific antibodies induced by the PPM nanovaccine in immunized mice, we next performed an antibody avidity assay, which measures the functional relevance of the produced antigen-specific antibodies. Urea was employed as a chaotropic agent for the elution of low-avidity antibodies upon incubation with the antigen-antibody complexes. The avidity index (%) of M278- and MOMP-specific IgG1 and IgG2b antibodies were determined using diluted sera (1:50, 1:100, 1:200 and 1:400). Our results (Figures 6A,B) reveal higher M278- and MOMP-specific IgG1 antibody avidities in the pre-challenge sera of PPM- as compared to the bare M278-immunized mice. Moreover, a Cm challenge did not impact the antibody avidity index. Conversely, an enhanced M278- and MOMP-specific IgG2b antibody avidity occurred in both mouse groups, albeit markedly higher in the PPM group (Figures 6C,D). Collectively, the avidity data shows that antigen-specific antibodies produced by PPM-immunized mice are more functional than those produced by the bare M278-immunized mice.

### Immune sera neutralization of *Chlamydia* infectivity *in vitro*

**Figure 7:**
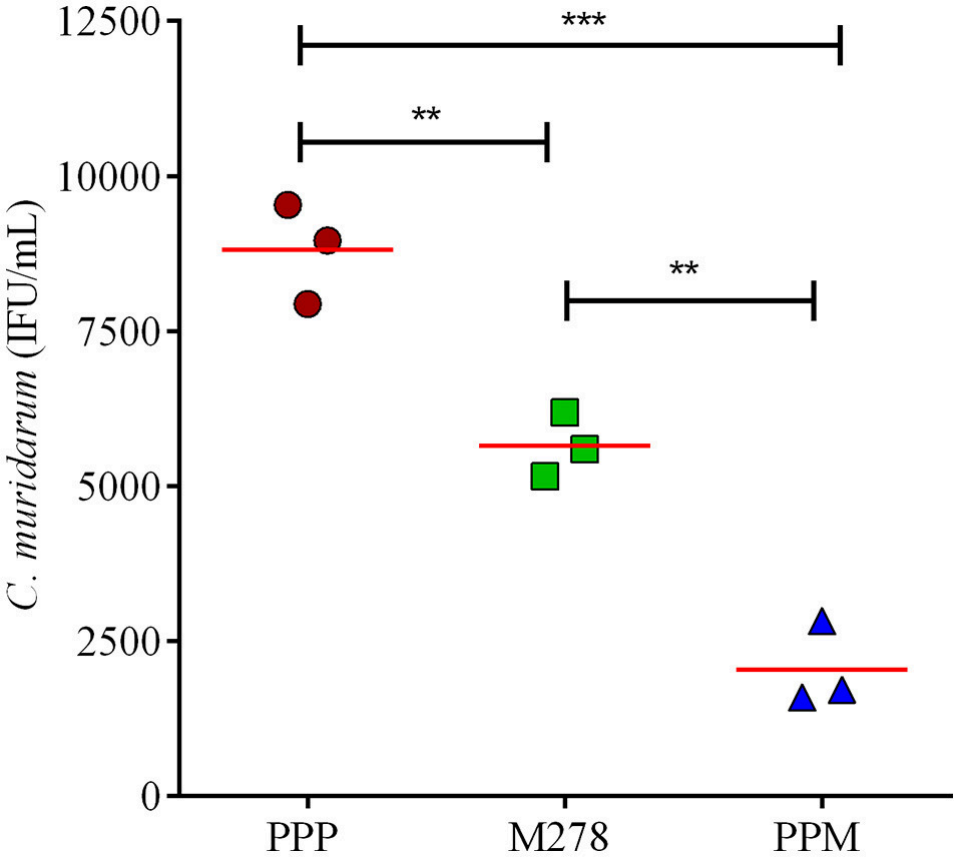
Serum-mediated *Chlamydia* infectivity of mouse fibroblasts. McCoy fibroblasts were infected with sera-bacteria inoculum in triplicates in a 96-well plate and incubated for 30 h. Cells were fixed and stained and inclusion forming units (IFU) were quantified by immunofluorescence microscopy. Results are shown as *C. muridarum* (IFU/mL) detected in cells exposed to pooled sera from immunized mice. Each symbol represents the mean of counts from 5 fields and 3 different wells; horizontal red line is the mean IFU/mL per group. Significance was considered at ***P < 0.001 and **P < 0.01. PPP (PLA-PEG-PBS immunized group); M278 (bare M278-immunized mice); PPM (PLA-PEG-M278)-immunized mice.

Since MOMP is known to induce neutralizing antibodies, we assessed the functionality of the produced antibodies in sera from PPP, bare M278, and PPM immunized mice to neutralize the infectivity of *Chlamydia* in McCoy fibroblasts. Fibroblasts were infected with Cm that had been pre-incubated with immune sera followed by incubation and quantification of IFUs. As depicted in Figure 7, the infectivity of cells treated with sera from PPM-immunized mice was significantly reduced (*P* < 0.001 to *P* < 0.01) respectively, by 77 and 64% as compared to those of the PPP- and bare M278-immunized mice. Conversely, the reduction in infectivity of cells treated with sera from the bare M278-immunized mice was only 36% in comparison to the PPP group. The results suggest that antibodies produced in the PPM immunized mice had stronger neutralizing activity against Cm as compared to bare M278 immunized mice. Our results highlight the neutralizing capacity of serum antibodies produced by PPM immunization, which inhibited *Chlamydia* infectivity of cells and their possible role in providing protection of mice against a chlamydial genital tract challenge.

## Discussion

Experiments with animal models of *Chlamydia* genital tract challenge have suggested that a vaccination strategy that could engender a sufficiently high mucosal immunity would be desirable to control the pathogen (28, 51, 52). Preferably, the vaccine delivery vehicle should be one that delivers the vaccine candidate without altering its conformation, prevents its degradation and ensures its slow and sustained release (53). Encapsulating antigens within polymeric nanoparticles facilitates their controlled release, depending on the matrix degradation rate, that allows better recognition and uptake by APCs, eventually leading to an enhanced immune response (35, 40). Biodegradable polymeric nanoparticles offers safety, flexibility of size manipulations, fabrication for targeted or passive delivery as well as controlled release of the encapsulated biomolecules (54–56) thus providing a novel approach for vaccine design with or without adjuvants (44).

Currently, biodegradable nanoparticles are being exploited for developing an efficacious vaccine against *Chlamydia* (6, 35, 36, 43, 44, 57); however, most of them are still in pre-clinical developmental stages (58). Amongst several available polymeric biodegradable nanoparticles, PLGA (6, 35), PLA-PEG (36) and the recently developed glycol-chitosan-coated lipid-polymer hybrid nanoparticles (57) have been validated to induce robust immune responses when used as a delivery system for *Chlamydia* vaccine candidates. The latest concept of surface charge-switching biodegradable nanoparticles (cSAPs) composed of poly (D, L-lactic-co-glycolic acid)-b-poly(L-histidine)-b-poly(ethylene glycol; PLGA-PLH-PEG) also has emerged as an exciting advancement in *Chlamydia* vaccine development (43). Here in the present study, we evaluated the capacity of our PPM nanovaccine to provide protection of female mice against a Cm genital tract challenge, and report our findings on the (1) protective efficacy provided by the nanovaccine in immunized mice and (2) elicited pre-and post-challenge protective adaptive immune responses in immunized mice.

Subcutaneous immunization with PPM protected mice against a vaginal challenge with Cm as compared to the bare M278 immunized mice, suggesting a critical role played by the PLA-PEG delivery system in affording mice the enhanced protection. The PLA-PEG co-polymeric nanoparticles have several unique properties (44, 59). PLA possesses low hydrophilicity, long degradation time, low drug loading (59), and inadequate interaction with cells (60). Contrastingly, PEG exhibits high hydrophilicity, phagocytic escape, resistance to immunological recognition, non-combination with serum proteins, low cytotoxicity, better interaction with biological surfaces, and high cell permeability (61, 62). Their co-polymerization forms a solid PLA core surrounded by PEG chains that is advantageous in improving the hydrophilicity, increased loading capacity, and a reduced burst effect (59). The PEG chains act as protein repellents and prevent protein degradation by proteases thereby facilitating extended biodegradation of the copolymer and the prolonged *in vivo* release of the encapsulated targets (44, 63). These attributes bolster PLA-PEG efficient delivery of vaccine candidates as observed here for *Chlamydia* and also for tumor-associated antigen (TAA) in cancer immunotherapy (64) and hepatitis B surface antigen (HBsAg) by provoking intense immune responses against the encapsulated antigens (63).

Undoubtedly, the self-adjuvanting property of the PLA-PEG nanoparticles, contributed to the protection afforded to mice by the M278 peptide against a Cm challenge. Peptide derivatives of MOMP (32, 38) are indeed effective in affording protection against a chlamydial challenge in animal models, but most require an adjuvant or a combination with other chlamydial antigens (21) to achieve protection. Other investigators also report that immunization with MOMP combined with adjuvants, such as modified microbial toxins (7, 16, 28), TLR agonists (30, 41), CpG plus Montanide ISA 720 VG (29, 49, 65) or liposomes (7) enhanced the protection in mice against challenge infections. A mucosal immunization with UV-Ct complexed with cSAPs and a TLR7/8 agonist conferred long-lived protection against *Chlamydia* in mice (43), further underscoring the significance of immunomodulatory adjuvants in enhancing *Chlamydia* protective immunity. In the present study, we did not include an exogenous adjuvant in our formulation mainly to test the self-adjuvanticity of the PLA-PEG nanoparticles as a delivery vehicle for the M278 peptide, and show that PPM alone significantly provided a level of protection in immunized mice by the reduced bacterial loads. However, complete protection of mice was not attained, which conjecturally could be due to either the small M278 peptide (aa 278-370) and/or the need for a mucosal adjuvant in the formulation to provide broader and more enhanced protection of mice. Overall, the ability of subcutaneous vaccination with PLA-PEG to potentiate mucosal immunity has not been reported, and this study provides the first evidence toward this end in our *Chlamydia* nanovaccine with slow releasing properties.

Activation and expansion of T-cells are essential in limiting intracellular pathogens via adaptive immune response mechanisms (6, 66, 67). The immune-potentiating property of PPM in mice was apparent by the up regulation of effector cytokines that facilitate CD4^+^ T-cell priming that is vital in effecting proliferative and effector properties of activating cells (68). The elevated levels of IFN-γ and IL-2 as produced by T-cells from PPM-immunized mice indicate that Th1 immune mechanisms were activated predominantly to produce a strong IFN-γ response; the most important acquired immunity correlate in *C. trachomatis* infections (4). For example, a synergistic role of IFN-γ and IL-2 is shown to provide protection against chlamydial genital tract infections (69). Apparently, IFN-γ inhibits chlamydial growth (37, 49, 50, 70) through stimulation of indoleamine-2,3-dioxygenase (IDO), which degrades tryptophan a key amino acid for *Chlamydia* survival (22, 71). Others report that IFN-γ upregulates iNOS genes in the genital epithelia to produce nitric oxide (NO), which mediates killing of *Chlamydia* (72). Although IL-2 does not participate directly in effector functions, its role in enhancing the expansion of effector T-cells and maintaining them in circulation is well documented (36, 69, 73). Reportedly T-cells co-expressing TNF-α/IL-2/IFN-γ elicit heightened protection against a genital chlamydial challenge, which provides additional evidence for the protective role of IFN-γ and IL-2 in chlamydial infections (17).

Of importance was the nanovaccine's triggering of robust CD4^+^ T-cells proliferation, and their differentiation into memory (CD44^high^ and CD62L^high^) and effector (CD44^high^ and CD62L^low^) phenotypes, thereby underscoring our recent findings (40). Results from animal models have demonstrated that immune mechanisms mediated by polyfunctional CD4^+^ T helper cells that co-secrete a number of Th1-cytokines like IFN-γ (4, 40, 43, 50) and TNF-α (4, 43, 50, 70) are important correlates of acquired immunity in *C. trachomatis* infections and are also associated with protection against re-infection. Additionally, CD4^+^ T-cells are reported to interact with antigen-specific B-cells to produce high-affinity long-lived memory and plasma cells (20). The high levels of IL-2 induced by PPM seemingly coincide with the CD4^+^ effector T-cells proliferative responses, as IL-2 is associated with proliferation and maintenance of effector T-cells. We can surmise that facilitation of PLA-PEG uptake by APCs for antigen processing and triggering of effector cytokines may have induced proliferation of chlamydia-specific CD4^+^ T-cells and directed their lineage toward memory and effector phenotypes. The role of tissue-resident memory T-cells in mediating protective immunity toward a *C. trachomatis* genital challenge has been reported (43); however, they were not investigated in the present study. Nonetheless, our findings are sufficient to indicate that PPM triggered the generation of antigen-specific memory in the nanovaccinated mice that presumably may have contributed to their protection against chlamydial infection.

It is well-established that humoral immunity also participates in limiting the spread of chlamydial infections and clearance during re-infection (7, 20), although the precise mechanistic role of antibodies are not well-understood (1). But, antibody-mediated neutralization and opsonization as well as antigen presentation to T-cells followed by receptor-mediated uptake are some possible mechanisms through which B-cells participate in protective immunity to re-infection (16, 22). In the present study, our nanovaccine effectively induced specific serum and mucosal antibody responses along with neutralizing antibodies against *Chlamydia*. The observed high IgG1 antibody titers in PPM-immunized mice are within agreement with our previous report (36) or other investigators that employed PLA-PEG as a delivery system for hepatitis B surface antigen (63), and also has been correlated with a protective immune response against *Chlamydia* (24, 32, 74). Although Th2 responses were dominant in the PPM-immunized mice, as indicated by lower Th1/Th2 antibody ratios, the bacterial challenge of mice skewed the pattern toward a Th1 dominant response evidently by the increased Th1/Th2 antibody ratios. This suggests that Th2 subsets were the primary responding T-cell subsets (32) stimulated by PPM. However, a vaginal challenge with Cm might have induced the Th1 T-helper subset to produce overall mixed antibody responses with a skew toward a high Th1/Th2 antibody ratio. Moreover, the protective efficacy of our nanovaccine might be mediated by neutralizing antibodies present in the sera of PPM-immunized mice.

Avidity measures the degree of affinity of generated antibodies against the specific antigen and affinity maturation of B-cells transforms into either memory B-cells or antibody-secreting plasma cells. Thus, an increased antibody avidity for an antigen reflects the stringency of these selective processes (75). Our avidity data shows that specific antibodies triggered by the nanovaccine are highly functional pre- and post-challenge. We observed that high avidity antigen-specific IgG1 and IgG2b antibodies were produced after PPM immunization, but bacterial challenge affected the IgG2b avidity and rendered them more functional and with higher responses against MOMP, possibly due to more recognition of the protein following a bacterial challenge, given that MOMP is abundantly expressed on the surface of the bacteria. The association of higher antibody avidity with greater anti-bacterial or anti-viral activities is well documented in *Haemophilus influenza* (76), *Streptococcus pneumonia* (77)*, Neisseria meningitides* (37) and HPV (75, 78) vaccine development studies. Also, investigators have reported a direct correlation of high avidity antigen-specific antibody to development and maintenance of long-term B-cell memory responses (79). The high antibody percent avidity reflects the anti-*Chlamydia* properties of the nanovaccine and its ability to develop B-cell memory responses.

Studies conducted on *C. trachomatis*-infected women (80) as well as murine models of *Chlamydia* (2, 20, 28) demonstrating an inverse relation between concentration of IgA and the endocervical bacterial load, reveal the importance of neutralizing IgA antibody in conferring protective immunity at mucosal surfaces. Our data show that antigen-specific mucosal IgA was triggered by PPM in immunized mice both pre- and post-challenge. This may suggest that the enhanced mucosal IgA neutralizes the bacterium by either preventing attachment to genital epithelial cells or inhibit their intracellular replication to afford significant protection against the pathogen. The heightened mucosal IgA antibody responses in PPM-immunized and challenge mice may be explained by the fact that MOMP comprises almost 60% of the total chlamydial outer membrane protein mass (1) and with it being highly immunogenic (6), the antibodies generated against the whole bacterium are expected to contain a large proportion of anti-MOMP antibodies. Therefore, upon bacterial exposure, the immunized animals induced anti-*Chlamydia* IgA antibodies, which were detected by testing the sera against MOMP.

Interestingly, PLA-PEG nanoparticles being comparable in size and shape to *Chlamydia* EBs (36) are promising candidates for developing a vaccine delivery system against *Chlamydia* as vaccines mimicking actual pathogens are emerging as an appealing approach in vaccine design (81). The EBs escape the endocytic-lysosomal pathway and alternatively follow the caveolin-endocytic route for their uptake and internalization (82, 83). Our recent study (40) has proved that the PLA-PEG nanoparticles carrying M278, also follow the same pathway, thus mimicking the natural chlamydial infection to activate innate and adaptive immune responses. The efficacy of PLA-PEG nanoparticles as trans-mucosal vaccine carriers using the model antigen TT (tetanus toxoid) has been demonstrated via earlier studies (84, 85) thereby providing an alternative of these being administered via the mucosal route, which is essentially a more desirable way to induce better immune protective capacity against *C. trachomatis* using our nanovaccine formulation.

In summary, our data reveal the protective efficacy afforded to mice by our PPM nanovaccine, and highlight the potency of PLA-PEG self-adjuvanting nanodelivery system. Protection of mice correlated with enhanced *Chlamydia*-specific CD4^+^ T-cell immune effector responses, a mix Th1/Th2 systemic and mucosal IgA antibody response, and neutralizing antibodies. The observed skewed serum and genital mucosa Th1 antibody after challenge may have contributed to protecting mice against the bacterial infection. Although, PPM provided significant protection of mice against a Cm challenge, there is still a need to optimize the vaccine to improve its protective efficacy. An area of improvement could, perhaps be the encapsulation of larger regions of MOMP with T- and B-cell epitopes, or full MOMP within PLA-PEG nanoparticles and employing a mucosal adjuvant such as the enterotoxin of *Escherichia coli* (LT), CPG or mucoadhesive polymers like glycol chitosan. Such an advanced formulation may bolster the protective efficacy of the nanovaccine by augmenting mucosal IgA as well as memory and effector T-cell responses. Our ongoing studies include modification and optimization of PPM, which may provide complete protection of mice against a chlamydial genital tract challenge, further propelling the vaccine developmental efforts against *Chlamydia*.

## Ethics statement

All studies were carried out in accordance with the Principles for Use of Animals, the Guide for the Care and Use of Laboratory Animals, the Provisions of the Animal Welfare Act, and other applicable laws and regulations concerning the humane care and use of research animals. The protocol (# IACUC040415) was approved by Alabama State University's Institutional Animal Care and Use Committee. Experimental care of the animals was conducted in compliance to minimize any discomfort or stress. Mice were sacrificed under CO_2_ inhalation and followed by cervical dislocation; this method of euthanasia is consistent with the recommendations of the American Veterinary Medical Association's Panel on Euthanasia.

## Author contributions

RV, RS, and SD contributed to the design of the experiments, performed the experiments, analyzed the data, and wrote the manuscript. SAD, GG, and SS read and critically edited the manuscript. VD designed, critically edited the manuscript, and coordinated the project. All authors read and approved the final manuscript.

### Conflict of interest statement

The authors declare that the research was conducted in the absence of any commercial or financial relationships that could be construed as a potential conflict of interest.
