# Supplementary material for: The Chlamydia M278 Major Outer Membrane Peptide Encapsulated in the Poly(lactic acid)-Poly(ethylene glycol) Nanoparticulate Self-Adjuvanting Delivery System Protects Mice Against a Chlamydia muridarum Genital Tract Challenge by Stimulating Robust Systemic and Local Mucosal Immune Responses
Source: Front Immunol. 2018 Oct 15;9:2369. doi: 10.3389/fimmu.2018.02369 (PMC6196261; doi:10.3389/fimmu.2018.02369)
Supplement: Supplementary file 1 [file Data_Sheet_1.docx]

*Supplementary Material*

The *Chlamydia* M278 major outer membrane peptide encapsulated in the poly(lactic acid)-poly(ethylene glycol) nanoparticulate self-adjuvanting delivery system protects mice against a *Chlamydia muridarum* genital tract challenge by stimulating robust systemic and local mucosal immune responses

Richa Verma^1#,^ Rajnish Sahu^1#^, Saurabh Dixit^1#^, Skyla A. Duncan^1^, Guillermo H. Giambartolomei^2^, Shree R. Singh^1^, Vida A. Dennis^1*^

^1^Center for NanoBiotechnology Research, Alabama State University, Montgomery, AL, United States.

^2^Instituto de Inmunología, Genética y Metabolismo (INIGEM). CONICET. Universidad de Buenos Aires, Argentina.

^#^Authors made equal contributions to the manuscript.

***Correspondence:** Vida A. Dennis, [vdennis@alasu.edu](mailto:vdennis@alasu.edu)





**Figure S1.** Cytokines production by T-cells from immunized mice challenged with *C. muridarum*. Groups of mice received three subcutaneous immunizations at two-week intervals with either bare M278 or PPM. Mice were challenged intravaginally with 1×10^5^ IFU (inclusion forming units) of *C. muridarum* three-weeks following the last immunization. Purified T-cells (1×10^6^) and APCs (1×10^6^) were stimulated with purified M278 (5 μg/mL) and incubated for 48 h at 37°C in a 5% CO_2_ humidified atmosphere. Cell-free supernatants were collected by centrifugation and used to quantify the cytokines (IL-6, TNF- α and IFN-γ) using specific ELISA. Each bar represents the mean ± standard deviation of triplicate samples. Significance was considered at ****P* < 0.001. PPP (PLA-PEG-PBS); M278 (bare M278-immunized mice); PPM (PLA-PEG-M278)-immunized mice


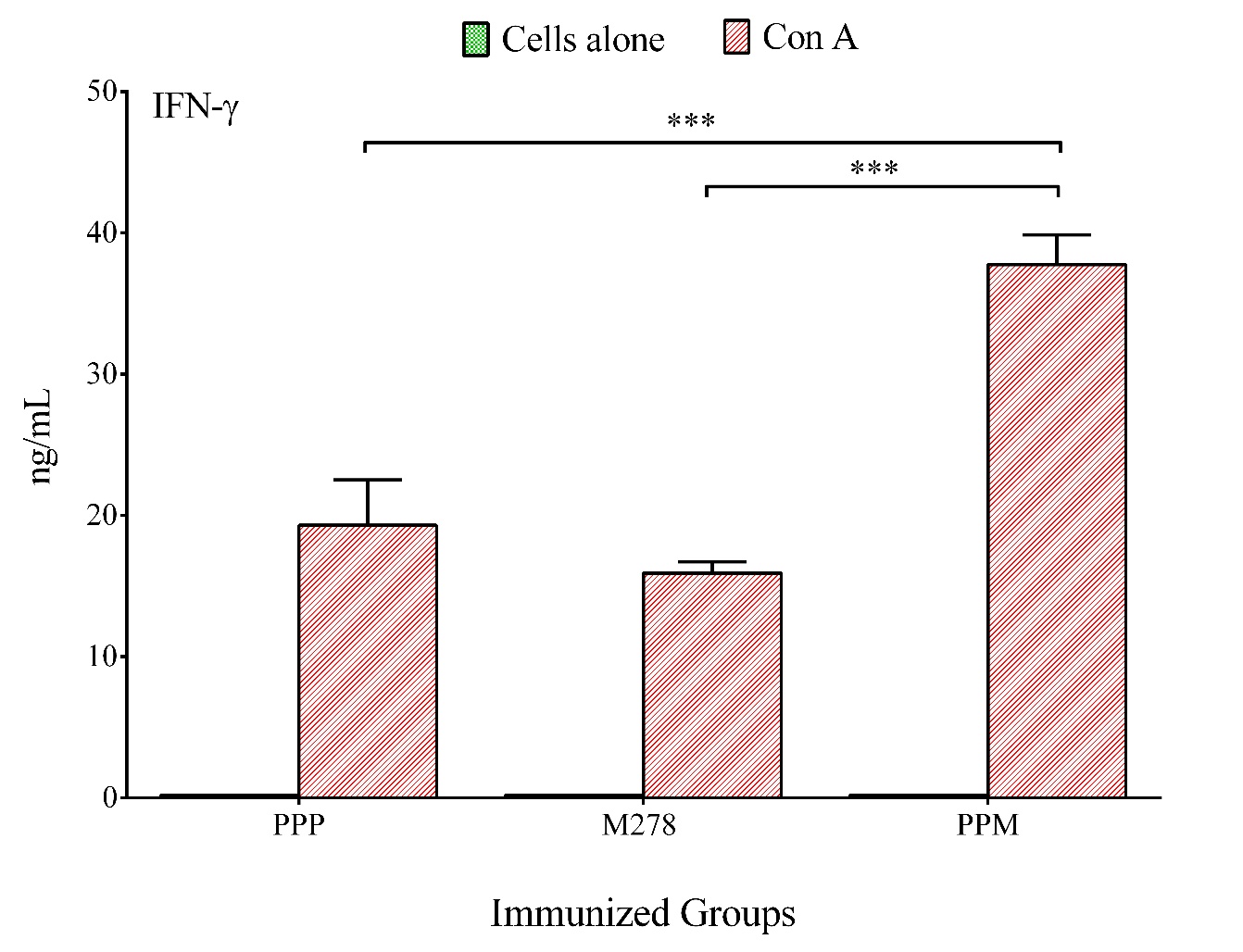


**Figure S2.** IFN-γ production by purified T-cells from immunized mice. Groups of mice received three subcutaneous immunizations at two-week intervals with either bare PPP, M278 or PPM. Purified T-cells (1×10^6^) and APCs (1×10^6^) were stimulated with Con A (5 μg/mL) and incubated for 48 h at 37°C in a 5% CO_2_ humidified atmosphere. Cell-free supernatants were collected by centrifugation and used to quantify IFN-γ using specific ELISA. Each bar represents the mean ± standard deviation of triplicate samples. Significance was considered at ****P* < 0.001. PPP (PLA-PEG-PBS); M278 (bare M278-immunized mice); PPM (PLA-PEG-M278)-immunized mice; Con A (Concanavalin A).
